# Determinants of Multi-Organ Morbidity in Neo-Transfusion-Dependent Thalassemia: A Cross-Sectional Analysis

**DOI:** 10.3390/jcm14186602

**Published:** 2025-09-19

**Authors:** Antonella Meloni, Paolo Ricchi, Laura Pistoia, Filomena Longo, Valerio Cecinati, Zelia Borsellino, Francesco Sorrentino, Elisabetta Corigliano, Michela Zerbini, Priscilla Fina, Ada Riva, Giuseppe Peritore, Vincenzo Positano, Alberto Clemente

**Affiliations:** 1 Bioengineering Unit, Fondazione G. Monasterio CNR-Regione Toscana, 56124 Pisa, Italy; positano@ftgm.it; 2 Unità Operativa Semplice Dipartimentale Malattie Rare del Globulo Rosso, Azienda Ospedaliera di Rilievo Nazionale “A. Cardarelli”, 80131 Napoli, Italy; paolo.ricchi@aocardarelli.it; 3 Unità Operativa Complessa Ricerca Clinica, Fondazione G. Monasterio CNR-Regione Toscana, 56124 Pisa, Italy; laura.pistoia@ftgm.it; 4 Dipartimento di Medicina Specialistica—Day Hospital della Talassemia e delle Emoglobinopatie, Azienda Ospedaliero-Universitaria Arcispedale “S. Anna”, 44124 Cona, FE, Italy; filomena.longo@ospfe.it; 5 Struttura Semplice di Microcitemia, Ospedale “SS. Annunziata”, 74123 Taranto, Italy; valerio.cecinati@asl.taranto.it; 6 Unità Operativa Complessa Ematologia con Talassemia, ARNAS Civico “Benfratelli-Di Cristina”, 90134 Palermo, Italy; zelia.borsellino@arnascivico.it; 7 Unità Operativa Semplice Day Hospital Talassemici, Ospedale “Sant’Eugenio”, 00143 Roma, Italy; sorrentino.francesco@aslrmc.it; 8 Ematologia Microcitemia, Ospedale San Giovanni di Dio—ASP Crotone, 88900 Crotone, Italy; krthal@libero.it; 9 Diagnostica per Immagini e Radiologia Interventistica, Ospedale del Delta, 44023 Lagosanto, FE, Italy; m.zerbini@ausl.fe.it; 10 Unità Operativa Complessa Diagnostica per Immagini, Ospedale “Sandro Pertini”, 00157 Roma, Italy; priscilla.fina@gmail.com; 11 Struttura Complessa di Radiologia, Ospedale “SS. Annunziata”, 74123 Taranto, Italy; ada.riva@asl.taranto.it; 12 Unità Operativa Complessa di Radiologia, ARNAS Civico “Benfratelli-Di Cristina”, 90134 Palermo, Italy; giuseppe.peritore@hotmail.it; 13 Department of Radiology, Fondazione G. Monasterio CNR-Regione Toscana, 56124 Pisa, Italy; clemente@ftgm.it

**Keywords:** neo-transfusion-dependent thalassemia, complications, iron overload

## Abstract

**Background:** This multicenter cross-sectional study aimed to assess the prevalence of vascular, hepatic, cardiac, endocrine, and bone complications and to identify factors associated with their occurrence in adult patients with neo-transfusion-dependent thalassemia (neo-TDT). **Methods:** A total of 140 adult neo-TDT patients (defined as receiving >4 transfusions/year; mean age 44.3 ± 12.1 years; 56.4% female) were retrospectively enrolled from the Extension–Myocardial Iron Overload in Thalassemia (E-MIOT) network. Iron overload (IO) was assessed by magnetic resonance imaging and complications were classified according to established clinical criteria. Logistic regression analyses were performed to investigate associations of complications with age, sex, splenectomy status, chelation therapy, hemoglobin < 9 g/dL, ferritin ≥ 1000 ng/mL, and hepatic, pancreatic, and cardiac IO. **Results:** Complications affecting fewer than 5% of patients—including leg ulcers, cirrhosis, thrombosis, heart failure, and hypoparathyroidism—were excluded from statistical analysis. Bone metabolism disorders were the most prevalent complications (68.6%), followed by impaired glucose metabolism (15.7%). The prevalence of other complications was: extramedullary hematopoiesis (EMH) 19.3%, pulmonary hypertension (PH) 7.1%, arrhythmias 12.1%, hypogonadism 11.4%, and hypothyroidism 15.0%. Male sex was independently associated with EMH (odds-ratio [OR] = 2.67; *p* = 0.027). Hepatic IO was the only significant predictor of PH (OR = 4.12; *p* = 0.047). Arrhythmias were strongly associated with older age (OR = 22.67; *p* < 0.0001), while both older age (OR = 4.42; *p* = 0.004) and pancreatic IO (OR = 7.40; *p* = 0.012) were independently associated with impaired glucose metabolism. No significant associations were identified for hypogonadism, hypothyroidism, or bone metabolism disorders. **Conclusion:** This study offers updated insights into the burden of complications in neo-TDT patients and highlights specific risk factors that may inform comprehensive, multidisciplinary surveillance strategies.

## 1. Introduction

Thalassemia refers to a group of inherited hemoglobin disorders caused by mutations affecting the synthesis of globin chains, leading to imbalanced hemoglobin production, ineffective erythropoiesis, chronic hemolytic anemia, and varying degrees of clinical severity [1,2,3,4]. Patients are broadly classified into two categories: transfusion-dependent thalassemia (TDT), requiring lifelong regular transfusions starting in early childhood, and non–transfusion-dependent thalassemia (NTDT), managed without regular transfusions thanks to the ability to maintain clinically acceptable hemoglobin levels [5]. NTDT includes conditions such as β-thalassemia intermedia, hemoglobin E/β-thalassemia, and α-thalassemia intermedia [6].

A proportion of patients with NTDT experience progressive clinical deterioration over time, ultimately necessitating regular blood transfusions in response to worsening anemia or the onset of complications resulting from intramedullary late erythroblastic destruction and tissue hypoxia such as bone pathology, leg ulcers, extramedullary hematopoiesis, cardiopulmonary complications, or thromboembolic events [7,8,9]. These patients are now classified as having neo–transfusion-dependent thalassemia (neo-TDT), a clinically distinct entity that differs from both NTDT and classical early-onset TDT [10]. In contrast to TDT patients, individuals with neo-TDT commence regular transfusion therapy during adolescence or adulthood, following prolonged exposure to anemia, tissue hypoxia, and ineffective erythropoiesis. Besides contributing to a unique pattern of morbidities, these underlying pathophysiologic processes drive increased gastrointestinal iron absorption and enhanced iron recycling, resulting in significant iron overload, predominantly hepatic [7,11,12,13,14]. So, the introduction of regular transfusion therapy in neo-TDT adds a significant transfusional iron burden to an already iron-loaded state. Recent magnetic resonance imaging (MRI) studies have provided important insights into the organ-specific patterns of iron deposition in patients with neo-TDT. While cardiac iron overload appears to remain relatively uncommon in this group [15,16], likely reflecting the shorter duration or lower intensity of transfusion exposure compared to classical TDT, pancreatic iron accumulation has emerged as a frequent and clinically significant finding [15,16]. Importantly, comparisons with NTDT patients revealed significantly higher levels of both cardiac and pancreatic siderosis in neo-TDT [15,16]. Of particular clinical relevance, the increased pancreatic iron accumulation in neo-TDT likely contributes to the heightened risk of glucose metabolism disturbances and related endocrine complications observed in this population [15].

The complication profile of neo-TDT remains poorly characterized in the literature. Most studies to date have either focused exclusively on NTDT [17,18] or grouped NTDT and neo-TDT together [19,20], potentially overlooking the specific risks associated with this transitioning phenotype. Given the unique disease trajectory and therapeutic exposure in neo-TDT patients, there is a critical need to better understand the factors driving the development of vascular, hepatic, cardiac, endocrine, and bone complications in this population. The aim of this multicenter cross-sectional study was to characterize the prevalence of these complications in adult neo-TDT patients and to identify the clinical and biochemical risk factors associated with their development. Understanding these associations may help guide earlier intervention strategies and inform personalized management approaches in this under-recognized and understudied patient group.

## 2. Materials and Methods

### 2.1. Study Population

The Extension-Myocardial Iron Overload in Thalassemia (E-MIOT) project is a nationwide collaborative network in Italy, involving 66 thalassemia centers and 15 MRI units. All MRI facilities adhere to standardized and validated protocols to maintain uniformity in data acquisition, analysis, and interpretation [21]. Clinical, laboratory, and imaging data are consistently collected and entered into a centralized web-based platform, which links all participating centers and enables integrated, standardized, and comprehensive documentation of each patient’s clinical profile.

From the cohort of adult (age ≥ 18 years) patients with hemoglobinopathies consecutively enrolled in the E-MIOT Network, we identified those with NTDT who had commenced regular transfusion therapy (administered every 3 weeks to 3 months) at least one year prior to their baseline MRI. A total of 140 neo-TDT patients met these criteria.

This study adhered to the ethical principles of the Declaration of Helsinki and has been approved by the ethics committees of all MRI centers involved. All patients provided written informed consent.

### 2.2. Iron Overload Assessment

MRI for the assessment of tissue iron overload was conducted on clinical 1.5 Tesla scanners from various manufacturers (GE Healthcare, Milwaukee, WI, USA; Philips Healthcare, Best, The Netherlands; Siemens Healthineers, Erlangen, Germany), all equipped with phased-array surface coils for signal reception. Imaging protocols included breath-hold techniques during end-expiration and electrocardiographic gating to minimize motion artifacts.

A single mid-axial slice of the liver [22], a minimum of five axial slices covering the entire pancreas [23], and short-axis slices of the left ventricle (LV) at basal, mid-ventricular, and apical levels [24] were obtained using multi-echo gradient-echo T2* sequences.

Experienced radiologists analyzed the T2* images using a dedicated, validated software (HIPPO MIOT^®^, Version 2.0, Consiglio Nazionale delle Ricerche and Fondazione Toscana Gabriele Monasterio, Pisa, Italy, Year 2015). T2* values were converted to R2* values using the formula R2* = 1000/T2*.

Hepatic R2* was measured within a standardized circular region of interest (ROI) [22] and then converted to liver iron concentration (LIC) through the application of an established calibration curve [25]. A MRI LIC value of 3 mg/g dry weight (dw) or higher was used to define hepatic iron overload [26]. Pancreatic iron quantification involved manually placing small ROIs in the head, body, and tail regions, carefully avoiding vessels, ducts, and artifacts from adjacent gastrointestinal gas [27]. The global pancreatic R2* value was determined by averaging the three regional R2* measurements. The upper normal limit for the global pancreatic R2* was set at 38 Hz [23]. The LV was segmented into 16 regions following the standard American Heart Association/American College of Cardiology (AHA/ACC) model [28], with R2* value assessed in each segment [24]. The global heart R2* value was calculated by averaging these segmental measurements. An upper normal limit of 31 Hz (equivalent to a T2* of 32 ms) was applied for cardiac iron assessment. This threshold was chosen over the more conservative 50 Hz (T2* = 20 ms) cut-off to improve sensitivity for the early detection of myocardial iron overload [29].

### 2.3. Laboratory and Instrumental Assessment

Biochemical analyses were performed at each participating thalassemia center using standardized clinical chemistry platforms, in accordance with established laboratory protocols. To ensure consistency, blood samples were collected in the morning following an overnight fast, centrifuged at 2500× *g* for 10 min, and analyzed promptly. Hemoglobin and ferritin were assessed at least six times per year per patient, and average values were used for analysis.

Hepatitis C virus (HCV) screening involved detection of anti-HCV antibodies via enzyme-linked immunosorbent assay (ELISA), followed by confirmation of active infection using reverse transcription polymerase chain reaction (RT-PCR) assays for HCV ribonucleic acid (RNA). Both qualitative and quantitative RT-PCR methods were utilized, with sensitivity thresholds capable of detecting viral loads above 50 IU/mL [30].

Glucose metabolism was evaluated using an oral glucose tolerance test (OGTT) [31] in all patients without a known diagnosis of diabetes. The test was conducted within three months of the MRI exam. Participants fasted for at least 12 h prior to testing. Fasting glucose and insulin levels were measured before administration of an oral glucose dose (1.75 g/kg body weight, up to a maximum of 75 g). Plasma glucose levels were then determined at one and two hours post-ingestion.

Bone mineral density (BMD) was assessed by dual-energy X-ray absorptiometry (DXA) at the lumbar spine (L1–L4) and right femoral neck. Results were expressed as T-scores (comparison to young adult reference population) and Z-scores (adjusted for age and sex), providing an evaluation of bone health status in relation to established normative values [32].

### 2.4. Diagnostic Criteria for Complications

Only active diseases at the time of MRI were considered.

Extramedullary erythropoiesis (EMH) was identified using computed tomography (CT) or MRI scans.

The diagnosis of leg ulcers was primarily based on a comprehensive medical history and a thorough clinical examination, including visual inspection and palpation of the affected area [33].

Liver cirrhosis was diagnosed by histology or based on clinical and laboratory findings combined with a positive radiologic result (i.e., CT, MRI, ultrasonography, endoscopy) [34].

Thromboembolic events include superficial venous thrombosis (SVT) and deep vein thrombosis (DVT). The diagnosis of SVT was primarily clinical, based on the presence of characteristic signs and symptoms. The diagnosis of DVT was confirmed by objective testing using ultrasound or venography.

Pulmonary hypertension (PH) was diagnosed if the trans-tricuspidal velocity jet was greater than 3.2 m/s [35].

Heart failure was diagnosed according to current guidelines, based on a combination of clinical symptoms (e.g., fatigue, dyspnea, and ankle swelling), physical signs, biomarker measurements, and imaging or other instrumental assessments [36]. Arrhythmias were confirmed by electrocardiographic documentation and the need for targeted treatment, and were classified following AHA/ACC guidelines [37].

The abnormalities in glucose metabolism were defined according to the ICET-A criteria [38]. Diabetes mellitus (DM) was defined as fasting glucose ≥ 126 mg/dL, the use of insulin or hypoglycemic agents, or 2-h plasma glucose ≥ 200 mg/dL; impaired glucose tolerance (IGT) as fasting plasma glucose < 126 mg/dL and 2-h plasma glucose between 140 mg/dL and 200 mg/dL; impaired fasting glucose (IFG) as fasting plasma glucose between 100 and 126 mg/dL.

Hypogonadotropic hypogonadism (HH) was defined by low blood levels of testosterone in males or 17β-estradiol in females in the presence of reduced pituitary gonadotropin levels [luteinizing hormone (LH) and follicle stimulating hormone (FSH)] [39]. Delayed puberty was identified in females by absent breast development, and in males by a testicular volume below 4 mL by the age of 16 years [39].

Hypothyroidism was diagnosed as follows: primary hypothyroidism was defined by elevated thyroid-stimulating hormone (TSH) levels with normal or reduced free thyroxine (FT4) levels, whereas central hypothyroidism was defined by reduced FT4 levels in the context of normal or reduced serum TSH [40].

Hypoparathyroidism was diagnosed based on low serum calcium levels, elevated serum phosphate, and either reduced parathyroid hormone (PTH) levels or PTH levels that were inappropriately normal relative to the degree of hypocalcemia [41].

Based on World Health Organization (WHO) criteria, a T-score of −1.0 or above was considered normal, a score between −1.0 and −2.5 was classified as osteopenia (low bone mass), and a T-score below −2.5 was diagnostic of osteoporosis [42].

### 2.5. Statistical Analysis

Data analysis was performed using SPSS version 27.0 statistical package (IBM Corp., Armonk, NY, USA).

Continuous variables were expressed as mean ± standard deviation (SD), while categorical variables were summarized using counts and percentages.

Normality of the continuous variables was evaluated using the Kolmogorov–Smirnov test.

Group comparisons were carried out using the independent-samples *t*-test for continuous variables with a normal distribution. In cases where continuous data did not follow a normal distribution, the Wilcoxon signed-rank test was applied. Categorical variables were compared using the chi-square test or Fisher’s exact test, as applicable.

Logistic regression analysis was conducted to examine the association between independent predictor variables and the binary outcome variable (presence of one complication). Odds ratios (ORs) and corresponding 95% confidence intervals (CIs) were computed to estimate the magnitude and direction of associations. Multivariate regression was performed using only variables that showed a *p* < 0.05 in the univariate analyses

All statistical tests were two-tailed, and a *p*-value < 0.05 was considered indicative of statistical significance.

## 3. Results

### 3.1. Characteristics of neo-TDT Patients

Demographic, clinical, hematological, and MRI characteristics and prevalence of vascular, hepatic, cardiac, endocrine, and bone complications in neo-TDT patients are described in Table 1.

Age showed a normal distribution according to the Kolmogorov–Smirnov test (*p* = 0.200). The patients had a mean age of 44.3 ± 12.1 years and were well distributed between the two sexes. Mean age at start of regular transfusions was 18.37 ± 19.09 years. More than two third of the patients were splenectomized and the mean age at splenectomy was 15.86 ± 10.47 years.

A hemoglobin <9 g/dL was found in the 13.1% of the patients.

Almost all patients (96.4%) received chelation therapy. The distribution of chelation regimens was as follows: 63% received deferasirox (mean dosage 22.24 ± 7.82 mg/kg body-weight/day if in dispersible tablets and 16.61 ± 4.75 mg/kg body-weight/day if in film-coated tablets), 16.3% desferrioxamine (mean dosage 35.06 ± 9.37 mg/kg body-weight/day with a frequency of 5.16 ± 0.92 days/week), 15.6% deferiprone (mean dosage 83.65 ± 18.73 mg/kg body-weight/day), and 5.2% were treated with a combination of two chelators.

The frequency of active HCV infection (defined by positive anti-HCV and HCV-RNA) or past HCV infection (resolved spontaneously or following antiviral treatment) was 41.4%.

While hepatic iron overload was found in less than 40% of patients, pancreatic iron overload was present in 70% of cases.

Bone metabolism disorders were the most common complication, affecting 68.6% of patients. EMH was identified in 27 (19.3%) patients, while leg ulcers, cirrhosis, and thromboembolic events each affected fewer than 5% of patients. Pulmonary hypertension was observed in 7.1% of cases.

The 12.9% of patients had active cardiac complications, primarily arrhythmias, with only one case of heart failure. Among the arrhythmias, the supraventricular arrhythmias (atrial fibrillation and atrial flutter) were the most common type (82.4%).

The 37.1% of the patients had at least one endocrine complication, including altered glucose metabolism, hypogonadism, hypothyroidism, and/or hypoparathyroidism. Disturbances of glucose metabolism were the most frequent endocrine disorder, found in 22 (15.7%) patients: 3 IFG, 12 IGT, and 7 DM.

### 3.2. Comparisons Between Complication and No-Complication Groups

Neo-TDT patients were stratified into two groups based on the presence or absence of specific morbidities and comparative analyses were performed between groups.

#### 3.2.1. EMH

Table 2 presents the comparison between neo-TDT patients without and with EMH. Male sex was associated with an increased prevalence of EMH. No significant differences were found in age, frequency of splenectomy and chelation, serum levels of hemoglobin and ferritin, and hepatic, pancreatic, and cardiac iron levels.

#### 3.2.2. PH

The comparison between patients without and with PH is shown in Table 3. Age, sex, and mean levels of hemoglobin and ferritin were comparable between the two groups. All patients with PH were splenectomized, but the prevalence of splenectomy was comparable between the two groups. The frequency of hepatic iron overload was significantly increased among patients with PH.

#### 3.2.3. Arrhythmias

Table 4 shows the comparison between patients without and with arrhythmias. Arrhythmias tended to be more prevalent among men, but the difference did not reach the statistical significance. The mean age was significantly higher in the group with arrhythmias. Hematochemical parameters, frequency of splenectomy and chelation, and organ-specific iron levels were comparable between the two groups.

#### 3.2.4. Endocrinopathies

Compared to patients with a normal glucose metabolism, patients with an altered glucose metabolism were significantly older and showed significantly higher pancreatic R2* values and a significantly increased frequency of pancreatic and cardiac iron overload (Table 5A). No difference was found in the other assessed parameters.

Pancreatic iron levels were significantly increased among patients with hypogonadism compared to patients without hypogonadism, while no significant differences were observed for any of the other parameters studied (Table 5B).

Patients without and with hypothyroidism were comparable for all the assessed parameters (Table 5C).

#### 3.2.5. Bone Metabolism Disorders

All the assessed parameters were comparable between patients without and with bone metabolism disorders (Table 6).

#### 3.2.6. Other Morbidities

Due to the limited number of affected patients, no comparisons were made between those without and with leg ulcers, without and with cirrhosis, without and with thrombosis, without and with heart failure, and without and with hypoparathyroidism.

Out of the 4 patients with cirrhosis, only one had hepatic iron overload. The remaining three patients with a normal MRI LIC had a history of HCV infection.

All patients with thromboembolic events had been previously splenectomized. Among them, only two had a serum ferritin level > 1000 ng/mL, while none had a haemoglobin < 9 g/dL.

The patient with heart failure had no myocardial iron overload and she also developed pulmonary hypertension.

### 3.3. Determinants of Complications

Table 7 shows the results of the binary logistic regression, conducted to identify the most significant independent predictors of each complication.

In the univariate regression analysis, male sex emerged as the only predictor of EMH (OR = 2.67, *p* = 0.027).

Only hepatic iron overload was associated with an increased risk of PH (OR = 4.12, *p* = 0.047).

The only significant risk factor for arrhythmias was age > 75th percentile (corresponding to 51.20 years) (OR = 22.67, *p* < 0.0001).

In the univariate regression analysis, increased age, cardiac iron overload, and pancreatic iron overload were significantly associated with an increased risk of alterations of glucose metabolism. In the multivariable regression analysis, the independent risk factors for an altered glucose metabolism were increased age (OR = 4.42, *p* = 0.004) and pancreatic iron overload (OR = 7.40, *p* = 0.012).

None of the considered variables emerged as a significant determinant of hypogonadism, hypothyroidism, or bone metabolism disorders.

## 4. Discussion

This study explored the determinants of complications in a contemporary cohort of NTDT patients who started transfusion therapy in late childhood or adulthood. Importantly, the hemoglobin levels reported in the tables reflect pre-transfusion values. Consequently, the actual mean hemoglobin achieved in this population is likely higher than that observed in classical NTDT patients or in certain previously reported mixed cohorts [17,43].

The low prevalence of leg ulcers, hepatic cirrhosis, and thrombotic complications prevented performing robust statistical analyses. However, descriptive findings confirm an association between liver cirrhosis and both HCV infection and iron overload [44,45], as well as a link between thrombotic events and splenectomy [46,47].

The prevalence of EMH was 19.3%, a rate similar to that reported among NTDT patients [18]. In our patient population, characterized by a late initiation of multiple blood transfusions, the presence of EMH may reflect the patient’s hematologic history and the extent of bone marrow dysfunction and compensation over time, or it may serve as a marker of ongoing hematopoietic stress, indicating a persistent residual demand for blood cell production despite transfusion therapy [48]. Male sex emerged as the only significant predictor of EMH. Our finding aligns with previous observations which indicated a sex-based disparity in the prevalence of EMH [49,50] and demonstrated that males had significantly increased pre-transfusion erythropoiesis and experienced a smaller post-transfusion reduction in erythropoiesis compared to females [51]. While biological factors (i.e., potential anabolic role of androgens in male) may contribute to these differences [52], it is also plausible that current transfusion regimens result in less effective suppression of erythropoiesis in males, indicating a relative degree of under-transfusion in this group.

Active PH was identified in the 7.1% of our patients. Direct comparison with NTDT populations is challenging due to the wide variability reported across studies, with prevalence ranging from 4.8% to 59%, depending on the characteristics of the cohort and the diagnostic methods employed [53,54,55]. Hepatic iron overload emerged as a significant predictor of PH, with an OR of 4.12. Several pathophysiological pathways may be implicated in this association. Hepatic siderosis is widely recognized as a surrogate marker of total body iron overload and reflects the cumulative effects of chronic transfusional iron accumulation and suboptimal chelation [26,56]. Excess iron has been shown to promote oxidative stress, endothelial dysfunction, and vascular remodeling—key processes in the pathogenesis of PH [57,58]. In addition, iron-induced liver dysfunction may contribute to altered hemodynamics, including the development of portopulmonary hypertension [59]. Moreover, in thalassemia, iron overload is often associated with ongoing ineffective erythropoiesis and hemolysis, which may further aggravate pulmonary vascular pathology through chronic hypoxia, nitric oxide scavenging, and a hypercoagulable state [60,61]. Therefore, the observed association between hepatic iron overload and PH may reflect not only direct iron-related vascular toxicity but also the broader systemic consequences of iron overload and disease severity. Our findings are in line with previous data from the OPTIMAL CARE study, which included both NTDT and neo-TDT patients and identified serum ferritin levels > 1000 ng/mL as an independent risk factor for PH development, although LIC values were not available in that cohort [43]. While splenectomy is a recognized risk factor for PH in thalassemia [54,55,62,63], the high proportion of splenectomized patients in our cohort may have limited our ability to detect an independent association between splenectomy and PH. Notably, all patients diagnosed with PH in our study had undergone splenectomy.

We did not observe an association between hemoglobin levels and either EMH or PH, likely due to the limited number of patients with hemoglobin levels below 9 g/dL. Nevertheless, our findings are consistent with previous studies that analyzed NTDT and neo-TDT patients as a combined cohort [20,43].

Arrhythmias—predominantly supraventricular—emerged as the most common cardiac complication in our cohort, whereas heart failure was infrequent, likely due to the low cardiac iron burden [64,65]. While MIO remains a key driver of heart failure in TDT, its role in atrial arrhythmogenesis appears substantially less relevant [64,66,67]. Consistent with this, our analysis found no association between left ventricular iron levels and supraventricular arrhythmia risk. Among the clinical variables assessed, age stood out as the only risk factor for arrhythmias, with an odds ratio of 22.67 for patients over 51.2 years. This observation is consistent with trends in the general population, where advancing age is a well-established risk factor for supraventricular arrhythmias, particularly atrial fibrillation [68,69]. Age-related supraventricular arrhythmias arise from a multifactorial and progressive atrial remodeling process involving impaired calcium handling, oxidative stress, mitochondrial and metabolic dysfunction, inflammation, fibrosis, and emerging mechanisms such as microbiome dysbiosis and proteolytic imbalance [69,70,71]. In neo-TDT patients, many of these pro-arrhythmic alterations seem occur earlier than in the general population, compounded also by the chronic high cardiac output characteristic of the disease [72,73,74,75], which imposes sustained hemodynamic stress on the atrial myocardium. With the growing life expectancy of thalassemia patients [76,77,78], early identification of arrhythmias is critical for optimizing long-term management, and a more comprehensive understanding of the underlying mechanisms may pave the way for personalized, stage-specific preventive and therapeutic interventions.

In our neo-TDT population, endocrine complications, particularly disturbances in glucose metabolism and hypothyroidism, were a significant and common feature and osteoporosis and osteopenia emerged as the major causes of morbidity.

Altered glucose metabolism was independently associated with both increased age and pancreatic iron overload, reaffirming findings from earlier studies involving patients with neo-TDT [15] as well as TDT [79,80]. The impaired insulin excretory function secondary to chronic iron overload in the pancreas is one of the main determinants of altered glucose metabolism in thalassemia [81,82,83].

No significant determinants were identified for hypothyroidism, hypogonadism, or bone metabolism disorders in our cohort. These findings contrast with those reported by Musallam et al., where elevated LIC was independently associated with increased risk of hypothyroidism, hypogonadism, and osteoporosis [20]. However, several key differences between the studies may account for this discrepancy. Notably, Musallam et al. analyzed a combined cohort of NTDT and neo-TDT patients and reported a higher mean LIC (8.4 ± 6.7 vs. 6.08 ± 10.66 mg/g dw in our study). Additionally, none of the patients in that study were receiving iron chelation therapy, whereas nearly all patients in our cohort were chelated. It is therefore possible that some individuals in our study may have experienced significant hepatic iron overload in the past, leading to irreversible endocrine damage, and were subsequently chelated to normalize or reduce iron levels [84,85]. This temporal disconnect may obscure associations between current LIC and endocrine complications.

The detailed characterization of the neo-TDT population in this study may gain increasing importance in the context of emerging therapies for NTDT, such as mitapivat, an activator of red cell pyruvate kinase, and luspatercept, an erythroid maturation agent [86]. Both have demonstrated substantial hemoglobin increases in randomized placebo—controlled clinical trials (ENERGIZE [87] and BEYOND [88]), but their long-term impact on disease-related complications remains unknown. The current data could therefore serve as a valuable a real-world benchmark to assess whether pharmacologically induced hemoglobin improvement leads to different patterns of organ morbidity compared to transfusion-based strategies.

### Limitations

One of the key limitations of our study lies in its retrospective design, which inherently restricts our ability to establish a clear timeline for the onset of complications in relation to the initiation of transfusion therapy. The absence of prospective data collection makes it challenging to draw causal inferences or to identify potential risk periods for specific adverse events. Furthermore, our assessment of tissue iron concentration and hematochemical parameters was based on data obtained at a single time point. Consequently, these measurements may not accurately capture the dynamic fluctuations in iron burden or biochemical markers over time, nor do they necessarily align with the clinical manifestation of individual complications.

With the exception of two patients carrying Hb Lepore in the heterozygous state and two patients with α-globin gene triplication, all patients in the cohort were diagnosed with β-thalassemia. Due to the very small number of patients with alternative genotypes, subgroup analyses regarding their association with complications were not feasible.

## 5. Conclusions

Our findings reveal key risk factors for complications in neo-TDT patients, underscoring the need for focused long-term monitoring. Male sex was identified as a significant risk factor for extramedullary hematopoiesis, while hepatic iron overload was associated with an increased risk of pulmonary hypertension. Advancing age emerged as a strong predictor of arrhythmias, and altered glucose metabolism was independently associated with both older age and pancreatic iron overload. Comprehensive, multidisciplinary surveillance is essential for the aging neo-TDT population to enable timely detection and management of these complications.

## Figures and Tables

**Table 1:** Demographic, clinical, hematological, and MRI data of neo-TDT patients.

	Neo-TDT Patients (N = 140)
Age (years)	44.30 ± 12.13
Females, N (%)	79 (56.4)
Age at start of regular transfusions (years)	18.37 ± 19.09
Splenectomy, N (%)	113 (80.7)
Chelated, N (%)	135 (96.4)
Mean hemoglobin (g/dL)	9.55 ± 0.58
Mean serum ferritin (ng/mL)	849.30 ± 908.51
Active/past HCV infection, N (%)	58 (41.4)
MRI LIC (mg/g dw)	6.08 ± 10.66
Hepatic IO, N (%)	54 (38.6)
Global heart R2* (Hz)	26.07 ± 6.49
Cardiac IO, N (%)	12 (8.6)
Global pancreas R2* (Hz)	98.61 ± 100.95
Pancreatic IO, N (%)	98 (70.0)
Extramedullary hematopoiesis, N (%)	27 (19.3)
Leg ulcers, N (%)	5 (3.6)
Cirrhosis, N (%)	4 (2.9)
Thromboembolic complications ^a^, N (%)	6 (4.3)
Superficial venous thrombosis, N (%)	3 (2.1)
Deep vein thrombosis, N (%)	3 (2.1)
Pulmonary hypertension, N (%)	10 (7.1)
Cardiac complications ^b^, N (%)	18 (12.9)
Heart failure, N (%)	1 (0.7)
Arrhythmias, N (%)	17 (12.1)
Endocrine complications ^c^, N (%)	52 (37.1)
Altered glucose metabolism, N (%)	22 (15.7)
Delayed puberty and hypogonadism, N (%)	16 (11.4)
Hypothyroidism, N (%)	21 (15.0)
Hypoparathyroidism, N (%)	1 (0.7)
Bone metabolism disorders, N (%)	96 (68.6)

TDT = transfusion dependent thalassemia, N = number, HCV = hepatitis C virus infection, MRI = magnetic resonance imaging, LIC = liver iron concentration, IO = iron overload. ^a^ Patients with superficial venous thrombosis, and/or deep vein thrombosis, and/or stroke. ^b^ Patients with heart failure and/or arrhythmias. ^c^ Patients with altered glucose metabolism, delayed puberty, hypogonadism, hypothyroidism, and/or hypoparathyroidism.

**Table 2:** Demographic, clinical, and MRI characteristics of neo-TDT patients stratified by the presence of EMH.

	No EMH (N = 113)	EMH (N = 27)	*p*-Value
Age (years)	43.94 ± 12.42	45.82 ± 10.89	0.455
Females, N (%)	69 (61.1)	10 (37.0)	0.024
Splenectomy, N (%)	90 (79.6)	23 (85.2)	0.599
Chelated, N (%)	110 (97.3)	25 (92.6)	0.246
Mean hemoglobin (g/dL)	9.54 ± 0.58	9.57 ± 0.58	0.888
Mean serum ferritin (ng/mL)	826.67 ± 863.59	938.08 ± 1081.55	0.927
MRI LIC (mg/g dw)	6.07 ± 11.49	6.13 ± 6.26	0.112
Hepatic IO, N (%)	42 (37.2)	12 (44.4)	0.485
Global pancreas R2* (Hz)	101.58 ± 107.77	86.18 ± 65.24	0.549
Pancreatic IO, N (%)	77 (68.1)	21 (77.8)	0.326
Global heart R2* (Hz)	26.00 ± 6.74	26.39 ± 5.45	0.130
Cardiac IO (32), N (%)	10 (8.8)	2 (7.4)	0.810

EMH = extramedullary hematopoiesis, N = number, MRI = magnetic resonance imaging, LIC = liver iron concentration, IO = iron overload.

**Table 3:** Demographic, clinical, and MRI characteristics of neo-TDT patients stratified by the presence of PH.

	No PH (N = 130)	PH (N = 10)	*p*-Value
Age (years)	44.04 ± 12.39	47.72 ± 7.65	0.198
Females, N (%)	74 (56.9)	5 (50.0)	0.747
Splenectomy, N (%)	103 (79.2)	10 (100.0)	0.209
Chelated, N (%)	126 (96.9)	9 (90.0)	0.256
Mean hemoglobin (g/dL)	9.56 ± 0.59	9.38 ± 0.28	0.203
Mean serum ferritin (ng/mL)	833.28 ± 907.82	1061.17 ± 944.87	0.404
MRI LIC (mg/g dw)	5.98 ± 10.94	7.41 ± 5.99	0.085
Hepatic IO, N (%)	47 (36.2)	7 (70.0)	0.045
Global pancreas R2* (Hz)	99.36 ± 102.32	88.91 ± 84.93	0.955
Pancreatic IO, N (%)	90 (69.2)	8 (80.0)	0.723
Global heart R2* (Hz)	26.19 ± 6.69	24.62 ± 2.38	0.808
Cardiac IO (32), N (%)	12 (9.2)	0 (0.0)	0.601

PH = pulmonary hypertension, N = number, MRI = magnetic resonance imaging, LIC = liver iron concentration, IO = iron overload.

**Table 4:** Demographic, clinical, and MRI characteristics of neo-TDT patients stratified by the presence of arrhythmias.

	No Arrhythmias (N = 123)	Arrhythmias (N = 17)	*p*-Value
Age (years)	42.48 ± 11.31	57.51 ± 9.55	<0.0001
Females, N (%)	73 (59.3)	6 (35.3)	0.061
Splenectomy, N (%)	97 (78.9)	16 (94.1)	0.194
Chelated, N (%)	118 (95.9)	17 (100)	0.397
Mean hemoglobin (g/dL)	9.55 ± 0.56	9.57 ± 0.71	0.970
Mean serum ferritin (ng/mL)	849.19 ± 925.54	850.18 ± 785.72	0.703
MRI LIC (mg/g dw)	6.44 ± 11.27	3.53 ± 3.32	0.304
Hepatic IO, N (%)	48 (39.0)	6 (35.3)	0.767
Global pancreas R2* (Hz)	97.76 ± 98.25	104.76 ± 121.99	0.735
Pancreatic IO, N (%)	87 (70.7)	11 (64.7)	0.585
Global heart R2* (Hz)	26.27 ± 6.82	24.66 ± 3.02	0.572
Cardiac IO (32), N (%)	11 (8.9)	1 (5.9)	0.673

N = number, MRI = magnetic resonance imaging, LIC = liver iron concentration, IO = iron overload.

**Table 5:** Demographic, clinical, and MRI characteristics of neo-TDT patients stratified by the presence of different endocrine disorders.

(A) Alterations of Glucose Metabolism			
	No Altered Glucose Metabolism (N = 118)	Altered Glucose Metabolism (N = 22)	*p*-Value
Age (years)	42.93 ± 11.85	51.66 ± 11.15	0.002
Females, N (%)	68 (57.6)	11 (50.0)	0.508
Splenectomy, N (%)	95 (80.5)	18 (81.8)	0.886
Chelated, N (%)	114 (96.6)	21 (95.5)	0.789
Mean hemoglobin (g/dL)	9.54 ± 0.56	9.61 ± 0.65	0.415
Mean serum ferritin (ng/mL)	857.69 ± 939.16	806.56 ± 751.15	0.587
MRI LIC (mg/g dw)	6.47 ± 11.48	3.99 ± 3.61	0.925
Hepatic IO, N (%)	46 (39.0)	8 (36.4)	0.817
Global pancreas R2* (Hz)	92.17 ± 96.08	133.15 ± 120.59	0.033
Pancreatic IO, N (%)	78 (66.1)	20 (90.9)	0.022
Global heart R2* (Hz)	25.89 ± 6.63	27.06 ± 5.72	0.273
Cardiac IO (32), N (%)	7 (5.9)	5 (22.7)	0.023
**(B) Hypogonadism**			
	**No Hypogonadism** **(N = 124)**	**Hypogonadism** **(N = 16)**	***p*-Value**
Age (years)	44.45 ± 12.79	43.14 ± 4.28	0.714
Females, N (%)	64 (54.8)	11 (68.8)	0.423
Splenectomy, N (%)	99 (79.8)	14 (87.5)	0.737
Chelated, N (%)	119 (96.0)	16 (100)	0.413
Mean hemoglobin (g/dL)	9.52 ± 0.59	9.72 ± 0.41	0.308
Mean serum ferritin (ng/mL)	862.18 ± 871.72	759.19 ± 1164.11	0.150
MRI LIC (mg/g dw)	6.27 ± 11.11	4.68 ± 6.22	0.612
Hepatic IO, N (%)	49 (39.5)	5 (31.3)	0.595
Global pancreas R2* (Hz)	95.19 ± 102.92	125.09 ± 82.08	0.010
Pancreatic IO, N (%)	82 (66.1)	16 (100.0)	0.003
Global heart R2* (Hz)	25.92 ± 6.19	27.29 ± 8.61	0.640
Cardiac IO (32), N (%)	9 (7.3)	3 (18.8)	0.142
**(C) Hypothyroidism**			
	**No Hypothyroidism** **(N = 119)**	**Hypothyroidism (N = 21)**	***p*-Value**
Age (years)	44.23 ± 12.69	44.74 ± 8.48	0.660
Females, N (%)	65 (54.6)	14 (66.7)	0.305
Splenectomy, N (%)	96 (80.7)	17 (81.0)	0.976
Chelated, N (%)	114 (95.8)	21 (100)	0.339
Mean hemoglobin (g/dL)	9.56 ± 0.55	9.45 ± 0.70	0.924
Mean serum ferritin (ng/mL)	789.46 ± 780.35	1240.01 ± 1475.83	0.883
MRI LIC (mg/g dw)	5.88 ± 10.43	7.25 ± 12.07	0.471
Hepatic IO, N (%)	46 (38.7)	8 (38.1)	0.961
Global pancreas R2* (Hz)	97.89 ± 100.65	102.72 ± 105.03	0.643
Pancreatic IO, N (%)	82 (68.9)	16 (76.2)	0.611
Global heart R2* (Hz)	26.14 ± 6.51	25.68 ± 6.51	0.589
Cardiac IO (32), N (%)	11 (9.2)	1 (4.8)	0.693

N = number, MRI = magnetic resonance imaging, LIC = liver iron concentration, IO = iron overload.

**Table 6:** Demographic, clinical, and MRI characteristics of neo-TDT patients stratified by the presence of bone metabolism disorders.

	No Bone Metabolism Disorders (N = 44)	Bone Metabolism Disorders (N = 96)	*p*-Value
Age (years)	44.85 ± 13.65	44.06 ± 11.43	0.914
Females, N (%)	21 (47.7)	58 (60.4)	0.160
Splenectomy, N (%)	32 (72.7)	81 (84.4)	0.105
Chelated, N (%)	42 (95.5)	93 (96.9)	0.649
Mean hemoglobin (g/dL)	9.39 ± 0.59	9.62 ± 0.55	0.094
Mean serum ferritin (ng/mL)	777.33 ± 891.36	882.02 ± 919.38	0.616
MRI LIC (mg/g dw)	5.88 ± 9.32	6.18 ± 11.26	0.383
Hepatic IO, N (%)	14 (31.8)	40 (41.7)	0.266
Global pancreas R2* (Hz)	95.01 ± 111.18	100.27 ± 96.48	0.183
Pancreatic IO, N (%)	29 (65.9)	69 (71.9)	0.475
Global heart R2* (Hz)	25.74 ± 6.42	26.23 ± 6.55	0.146
Cardiac IO (32), N (%)	5 (11.4)	7 (7.3)	0.517

N = number, MRI = magnetic resonance imaging, LIC = liver iron concentration, IO = iron overload.

**Table 7:** Univariate and multivariate binary logistic regression analysis showing factors associated with each considered complication.

	Univariate Regression Analysis		Multivariate Regression Analysis	
	OR (95% CI)	*p*-Value	OR (95% CI)	*p*-Value
EMH				
Age > 75th percentile	1.67 (0.67–4.18)	0.269		
Male sex	2.67 (1.12–6.35)	0.027	2.67 (1.12–6.35)	0.027
Splenectomy	1.47 (0.46–4.67)	0.514		
Chelation therapy	0.34 (0.05–2.15)	0.252		
Hemoglobin < 9 g/dL	0.84 (0.22–3.17)	0.795		
Ferritin ≥ 1000 ng/mL	0.88 (0.32–2.42)	0.800		
Hepatic IO	1.35 (0.58–3.16)	0.486		
Pancreatic IO	1.64 (0.61–4.40)	0.329		
Cardiac IO	0.82 (0.17–4.00)	0.810		
**PH**				
Age > 75th percentile	1.31 (0.32–5.38)	0.705		
Male sex	1.32 (0.37–4.79)	0.671		
Splenectomy	-			
Chelation therapy	0.29 (0.03–2.83)	0.284		
Hemoglobin < 9 g/dL	0.72 (0.08–6.09)	0.765		
Ferritin ≥ 1000 ng/mL	2.60 (0.65–10.35)	0.175		
Hepatic IO	4.12 (1.02–16.69)	0.047	4.12 (1.02–16.69)	0.047
Pancreatic IO	1. 79 (0.36–8.75)	0.479		
Cardiac IO	-			
**Arrhythmias**				
Age > 75th percentile	22.67 (5.98–85.92)	<0.0001	22.67 (5.98–85.92)	<0.0001
Male sex	2.68 (0.93–7.71)	0.068		
Splenectomy	4.29 (0.54–33.86)	0.167		
Chelation therapy	-			
Hemoglobin < 9 g/dL	0.48 (0.06–3.93)	0.495		
Ferritin ≥ 1000 ng/mL	1.24 (0.36–4.28)	0.730		
Hepatic IO	0.85 (0.29–2.46)	0.767		
Pancreatic IO	0.76 (0.26–2.21)	0.612		
Cardiac IO	0.64 (0.08–5.27)	0.675		
**Alterations of glucose metabolism**				
Age > 75th percentile	3.10 (1.21–8.00)	0.019	4.42 (1.59–12.31)	0.004
Male sex	1.36 (0.55–3.39)	0.509		
Splenectomy	1.09 (0.34–3.53)	0.886		
Chelation therapy	0.74 (0.08–6.92)	0.789		
Hemoglobin < 9 g/dL	0.66 (0.14–3.13)	0.600		
Ferritin ≥ 1000 ng/mL	1.66 (0.60–4.56)	0.326		
Hepatic IO	0.89 (0.35–2.29)	0.817		
Pancreatic IO	5.13 (1.14–23.05)	0.033	7.40 (1.55–35.46)	0.012
Cardiac IO	4.66 (1.33–16.38)	0.016		
**Hypogonadism**				
Age > 75th percentile	0.18 (0.02–1.39)	0.099		
Male sex	0.55 (0.18–1.68)	0.296		
Splenectomy	1.77 (0.38–8.29)	0.470		
Chelation therapy	-			
Hemoglobin < 9 g/dL	-			
Ferritin ≥ 1000 ng/mL	0.67 (0.18–2.51)	0.551		
Hepatic IO	0.69 (0.23–2.12)	0.524		
Pancreatic IO	-	0.997		
Cardiac IO	2.95 (0.71–12.28)	0.137		
**Hypothyroidism**				
Age > 75th percentile	0.93 (0.31–2.75)	0.891		
Male sex	0.60 (0.23–1.59)	0.308		
Splenectomy	1.02 (0.31–3.32)	0.976		
Chelation therapy	-			
Hemoglobin < 9 g/dL	2.01 (0.58–6.99)	0.272		
Ferritin ≥ 1000 ng/mL	1.19 (0.39–3.66)	0.753		
Hepatic IO	0.98 (0.38–2.54)	0.961		
Pancreatic IO	1.44 (0.49–4.24)	0.504		
Cardiac IO	0.49 (0.06–4.02)	0.507		
**Bone metabolism disorders**				
Age > 75th percentile	1.00 (0.44–2.28)	1.000		
Male sex	0.59 (0.29–1.23)	0.161		
Splenectomy	2.03 (0.86–4.79)	0.109		
Chelation therapy	1.48 (0.24–9.17)	0.676		
Hemoglobin < 9 g/dL	0.62 (0.22–1.75)	0.362		
Ferritin ≥ 1000 ng/mL	1.48 (0.59–3.65)	0.398		
Hepatic IO	1.53 (0.72–3.25)	0.268		
Pancreatic IO	1.32 (0.62–2.84)	0.475		
Cardiac IO	0.61 (0.18–2.05)	0.428		

OR = odds ratio, CI = confidence intervals, EMH = extramedullary hematopoiesis, IO = iron overload, PH = pulmonary hypertension.

## Data Availability

The data presented in this study are available on request from the corresponding author. The data are not publicly available due to privacy.
